# Evaluation of Material Appearance Under Different Spotlight Distributions Compared to Natural Illumination †

**DOI:** 10.3390/jimaging5020031

**Published:** 2019-02-21

**Authors:** Takashi Yamazoe, Tomohiro Funaki, Yuki Kiyasu, Yoko Mizokami

**Affiliations:** 1 Institute for Global Prominent Research, Chiba University, 1-33, Yayoicho, Inage Ward, Chiba-shi, Chiba 263-8522, Japan; 2 Department of Imaging Sciences, Graduate School of Science and Engineering, Chiba University, 1-33, Yayoicho, Inage Ward, Chiba-shi, Chiba 263-8522, Japan; 3 Graduate School of Engineering, Chiba University, 1-33, Yayoicho, Inage Ward, Chiba-shi, Chiba 263-8522, Japan

**Keywords:** material perception, material appearance, lighting distribution, illumination diffuseness

## Abstract

Solid-state lamps including Organic Light Emitting Diode (OLED) lighting could facilitate a wide variety of lighting conditions by controlling the spectral power distribution and the spatial distribution of the light source. The appearance of the surface of an object is significantly influenced by the lighting conditions and the constituent materials of the objects. Therefore, appearance of objects may appear to be different from expectation. Lighting condition leads to important part of accurate material recognition. We investigate whether it is possible to determine the lighting condition that results in the intended material appearance by the evaluation of this parameter under different lighting distributions compared to natural illumination. The viewing conditions of three spotlight sizes and three illuminance levels were investigated. The participants selected the viewing condition for which the appearance of fruits and vegetable food samples was the closest to the impressions learned from observing and freely holding these objects under natural reference illumination. Participants also evaluated their impressions of stimuli in each viewing condition by responding to twelve questions. The results show that the wide spotlight size condition with higher diffuseness of the illumination was selected more frequently than the narrow spotlight conditions. This suggests that the diffuseness of illumination influences the appearance of the object’s material. The results of seven-point scales suggest that their impression of stimuli was influenced by the surface properties of the objects as well as the lighting distributions. It was suggested that it is possible to set an appropriate lighting condition to facilitate material appearance similar to the expected appearance under natural illumination.

## 1. Introduction

The recent development of new solid-state lamps including OLED lighting would facilitate a wide variety of lighting conditions by controlling the spectral power distribution and the spatial distribution of the light source. For example, OLED lighting could be in the form of a surface light source with strong diffuseness. There are a variety of other ways of changing lighting distributions such as using spotlights that change the size of an illuminated area by controlling the separation between a lens and a light source. This kind of change in lighting distribution influences the lighting conditions such as the properties of shadow and overall diffuseness.

The appearance of an object’s surface could be significantly influenced by the lighting conditions and the constituent materials of the object. There is much research activity on color rendering of lighting and the influence of lighting on color appearance. A technical report on new color fidelity index for accurate scientific use has been recently published [1] and other evaluation methods are under consideration at the International Commission on Illumination (CIE). However, the influence of the diffuseness of lighting on material appearance has not been systematically analyzed.

The material and texture of objects provide important information that determines object impression. Material perception can be mainly classified into visual and tactile sensation. The combination of this information forms the basis for the recognition and impression of an object. Moreover, we typically experience good material perception via visual observation without touching. However, this perception is not perfect and could change under different lighting conditions. Therefore, it is important to realize a desirable material impression under artificial lighting. One important characteristic of a desirable material impression is the fidelity of material appearance corresponding to color fidelity, which evaluates the similarity of color appearance under artificial lighting compared to natural illumination. It would be useful if it was possible to identify a lighting condition that realizes accurate material appearance.

The perception of an object’s surface is influenced by color (including hue, value, and chroma), material property (including diffuse and specular reflection, scattering, and transparency) and the surface roughness of the object [2]. The random subsurface scattering of light and the emerging light ray distributed in a wide range of directions results in a matte appearance of the surface [3]. A previous study on computer-generated graphics established that appearance depends on viewpoint, illumination and the scale at which texture is observed [4]. This suggests that illumination, viewpoint, and surface conditions are important factors with respect to material perception. It was shown that both lightness and glossiness ratings were well correlated with the skewness of the luminance histogram [5]. The histogram of a gloss surface has a positive skewness and that of a matte surface has a negative skewness. Although image statics partially explain gloss and lightness perception, it was reported that the derivation of surface and material properties requires a photo-geometric analysis [6] and the perception of gloss can be understood as a direct consequence of image properties that covary with surface geometry and the illumination field [7]. It has been reported that our perceptual qualities are well-defined, distinct, and systematically related to material class membership [8]. However, material categories could be confused when materials are represented as degraded gray images [9,10]. In the cases where the correct perception of the light field is important, more emphasis should be placed on the realism of the global properties of the light field such as direction and diffuseness. In computer-generated graphics, the global properties of the light field such as the mean direction and directedness (or diffuseness) are crucial for light field perception. It has been reported that even in the images of complex objects, the perception of material and illumination were basically confounded [11]. A previous study showed that users were able to edit light fields using their tested interface and tools, even in the presence of imperfect depth [12]. The texture perception of roughness would be correlated to the global properties of the light field. Roughness constancy could fail due to a change in viewpoint as well as confound changes in surface roughness with changes in illumination [13]. It was shown that variations in the spatial structure of rather simple illumination influences perceived glossiness [14]. Moreover, material appearance is more diagnostic for materials than for lightings, causing asymmetric perceptual confounds [15]. Previous research suggests that texture perception is generated by the interaction between the optical condition associated with an object’s surface and the lighting environment. However, it has been suggested that the estimation of surface reflectance did not require knowledge of the specific conditions of illumination and we use assumptions based on our experience about the statistics of real-world illumination to estimate surface reflectance [16]. It was also reported that the assumptions of human observers about lighting diffuseness were well matched to the diffuseness of lighting in real-world scenes, suggesting that human vision is attuned to the diffuseness levels of natural lighting conditions [17]. It has also been reported that the diffusivity of lighting influenced the appearance of an object’s surface including glossiness and roughness [18], however, the color appearance was stable [19]. The diffuseness and direction of Illumination also influenced the appearance of textiles [20]. It was reported that there is an effect of material constancy among the different distribution of specular and diffuse reflection, but no obvious gloss constancy when observing only the distribution of specular reflection. This is because the shape and size of light sources have a significant influence on gloss perception, in research using printed paper objects [21]. These investigations suggest that the distribution and diffuseness of lighting can be a strong factor of material perception. The influence of illumination on the appearance of objects has been investigated and should be investigated further. However, it is not clear which type of lighting conditions are adequate to realize the appropriate appearance reproduction of materials.

In the present study, we investigate whether it is possible to establish a lighting condition to facilitate appropriate material appearance by comparing subjective evaluations under different lighting distribution with those under natural illumination. There is a wide variety of lighting conditions, changing light fields, and diffuseness. As such, it is difficult to test all conditions. Here, we focus on the effect of lighting with different spotlight sizes on the appearance of vegetables and fruits. We encounter these types of lighting conditions in shops, home and restaurants in our daily life and sometimes their appearance and impression are quite different from what we expect or prefer. Vegetables and fruits are objects with which we are familiar and their appearance is also important in our daily life. It would be useful to determine whether it is possible to evaluate the difference in naturalness or impression of objects under these lighting conditions and to identify an ideal lighting condition that exhibits their natural appearance.

## 2. Methods

A Light Emitting Diode (LED) lighting system to control spotlight size and intensity was constructed to evaluate the influence of lighting distribution to material appearance. We compared the appearance of fruit and vegetable food samples under different lighting conditions to the material impression of those objects under natural light. In a reference phase, participants held and observed stimulus objects under natural illumination and evaluated the impression of the objects’ appearance. Then, the participants visually evaluated the appearance of objects under nine viewing conditions (three spotlight conditions and three illuminance conditions) against the material impression of the objects under natural illumination.

### 2.1. Apparatus and Stimuli

**Figure 1:**
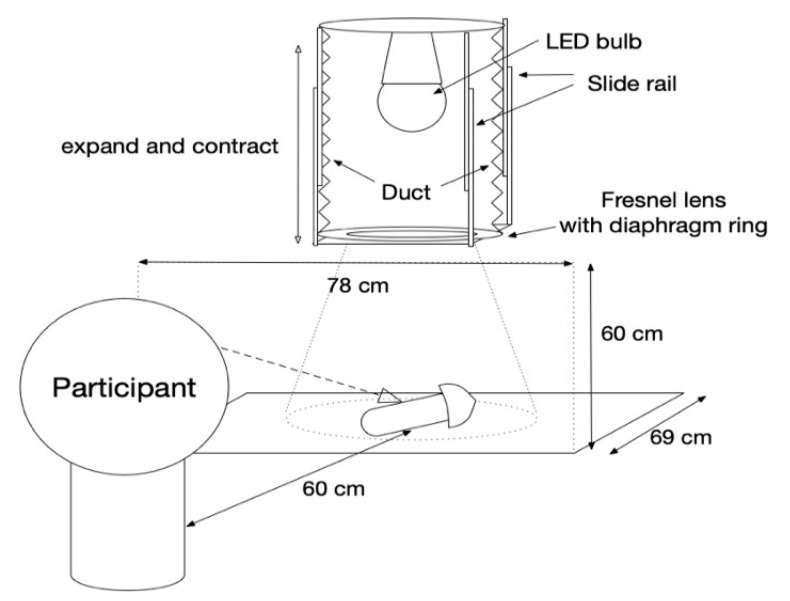
Arrangement of the experimental booth with a light emitting diode (LED) lighting system, and a stimulus.

A viewing booth was placed in a dark room and an LED lighting control system was set up on the ceiling of the viewing booth as shown in Figure 1. Participants viewed the booth through a window but they were not able to directly see the LED lighting.

**Figure 2:**
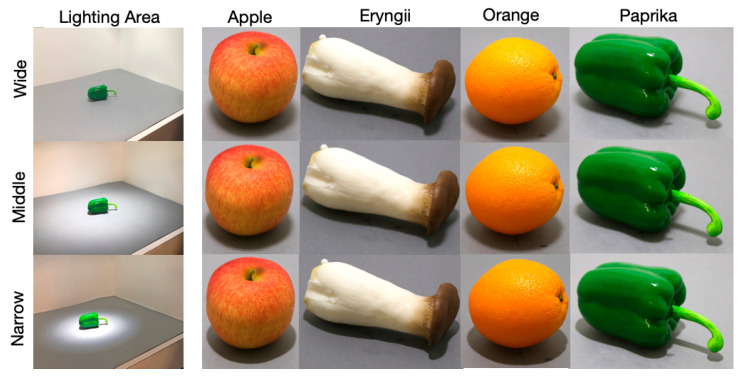
Spotlight size and the appearance of each stimulus. Wide size spotlight has high diffuseness, Middle size has moderate diffuseness, and Narrow size has low diffuseness.

Four kinds of food samples including Orange, Apple, Eryngii, and Paprika, were used as stimuli. These stimuli were selected to have a variety of combinations of matte-gloss (micro-texture) and smooth-rough (macro-texture) surfaces. Orange had a gloss and rough surface whereas Apple had a matte and smooth surface. Eryngii had a matte and rough surface whereas Paprika had a gloss and smooth surface. The stimuli are shown in Figure 2 for different spotlight sizes. Three spotlight sizes and three illuminance conditions were combined to establish nine viewing conditions.

**Figure 3:**
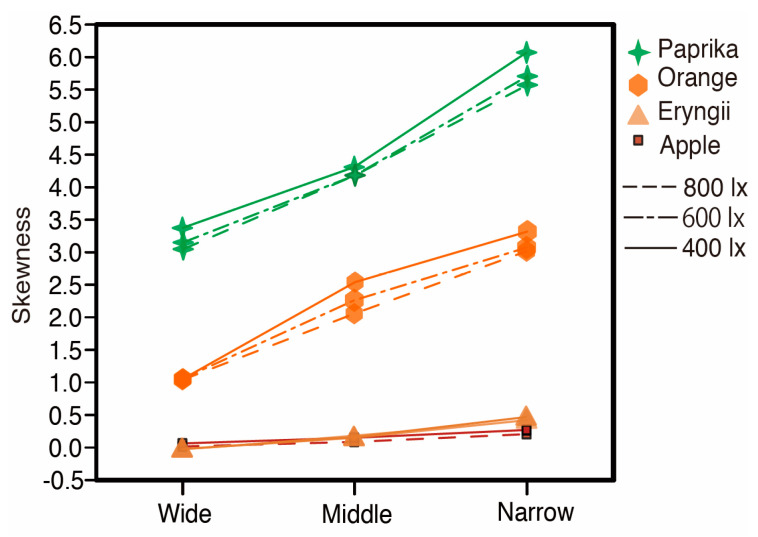
Relation between the skewness of the luminance histogram and spotlight size under three illuminance levels.

**Figure 4:**
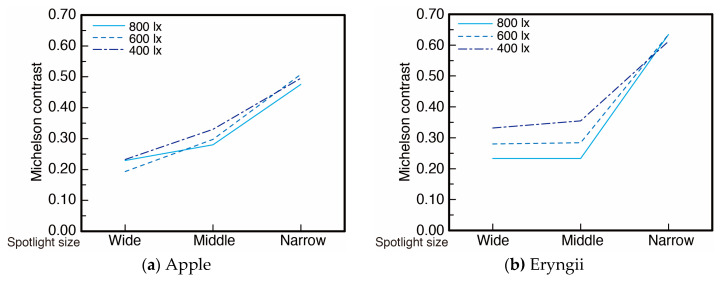

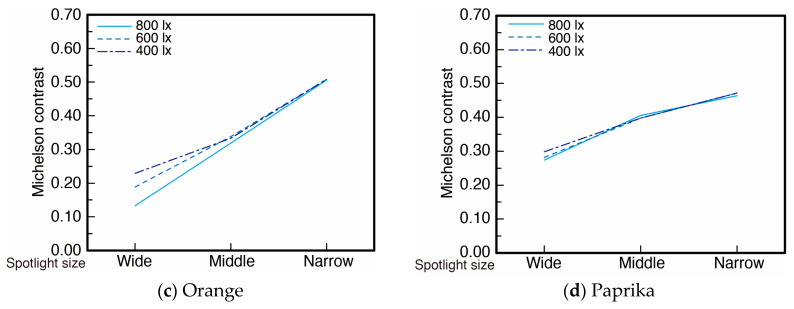
Relation between the Michelson contrast of luminance on samples and spotlight size under three illuminance levels for (**a**) Apple, (**b**) Eryngii, (**c**) Orange, (**d**) Paprika.

The LED lighting control system consisted of an LED bulb, a Fresnel lens, diaphragm rings, slide rails, and a flexible duct. An LED bulb (Panasonic LDA7DHEW2, CCT 6500 K, Ra 80) was used as a light source. The Fresnel lens and the slide rails were used for spotlight control. The four slide rails were able to guide the Fresnel lens to move up and down to change the distance between the Fresnel lens and the LED bulb. The spotlight size was controlled by changing this distance. The diaphragm rings controlled illuminance. We tested three levels of spotlight size; narrow, middle, and wide. The change in the size influenced the diffuseness condition of the lighting. Therefore, we calculated a diffuseness metric based on Cuttle’s vector/scalar illumination ratio [22,23]. We measured the diffuseness of the illumination at the position of a stimulus based on a cubic illuminance measurement. The illuminance of six directions (tilt angle +35° with rotate angle 0° (*E*(u+)), 120° (*E*(v+)), 240° (*E*(w+))), and tilt angle −35° with rotate angle 60° (*E*(w−)), 180° (*E*(u−)), 300° (*E*(v−)) was measured to calculate vector illuminance (|*E*|) and scalar illuminance (*E*_sr_). Diffuseness (*D*_Cuttle_ = 1 − (|*E*|/*E*_sr_)/4) can be specified from cylindrical illuminance and the working plane illuminance. The diffuseness was approximately 0.44 for a wide spot size, 0.34 for a middle spot size, and 0.17 for a narrow spot size condition as shown in Table 1. The relation between spotlight size and the skewness of luminance histogram of the samples under the three illuminance levels are shown in Figure 3. The relation between the spotlight size and the Michelson contrast of luminance in a sample region under three illuminance levels are shown in Figure 4. The luminance histogram and contrast were obtained from the measurement data of the samples using a two-dimensional luminance colorimeter (Konica Minolta CA-2000, resolution 980 × 980 pixels). The region of measurement for Apple, Eryngii, Orange, and Paprika was 121 cm^2^ (233,148 pixels, 5.82 pixel/mm), 126 cm^2^ (134,481 pixels, 5.07 pixel/mm), 116 cm^2^ (186,867 pixels, 5.34 pixel/mm), and 128 cm^2^ (199,050 pixels, 6.06 pixel/mm), respectively. Both image statistics changed corresponding to spotlight size.

Three illuminance levels (800 lx, 600 lx, and 400 lx) were also tested to examine the influence of the overall lighting level on the material impression of objects. We used the appearance of stimuli under natural illumination as a reference for fidelity evaluation, but it was difficult to realize a high illuminance environment (>1990 lx) in our lighting system. Three illuminance levels were tested to examine the influence of illuminance on fidelity evaluation and to confirm that the result was not determined solely by the illuminance difference between the test and reference environments.

**Figure 5:**
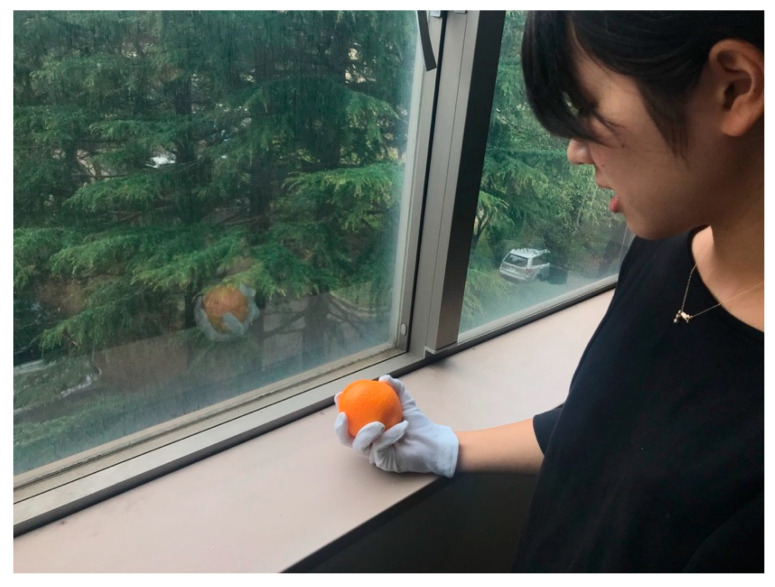
Natural illumination condition (next to a north window).

Natural illumination conditions were used during the referencing phase of the experiment to establish the criterion of the object’s material appearance. The standard illumination condition for material evaluation corresponding to that for color fidelity evaluation using a blackbody and daylight as a reference has not been established. Therefore, we decided to use a condition with free observation under natural illumination from a north-facing window as a reference because we normally use visual and tactile sensations to establish a firm recognition of materials. Moreover, natural illumination is the ideal lighting for recognizing objects in a similar manner to color evaluation. We chose a natural illumination condition in which illuminance and geometry largely change since we are usually able to establish “a stable and accurate object recognition (or memory)” not by observing under one particular viewing condition but by observing and touching under a variety of viewing conditions. Participants observed food samples illuminated by a north-facing window as shown in Figure 5. They were able to hold and rotate food samples to view them from different angles. The diffuseness (*D*_Cuttle_) of natural illumination varied from 0.501 to 0.574. These values represent relatively high diffuseness and the illuminance was varied from 1990 lx to 7010 lx. Although there are significant variations of illuminance and diffuseness in natural illumination, the results of evaluations under this type of illumination did not show significant differences.

### 2.2. Subjective Evaluation

**Figure 6:**
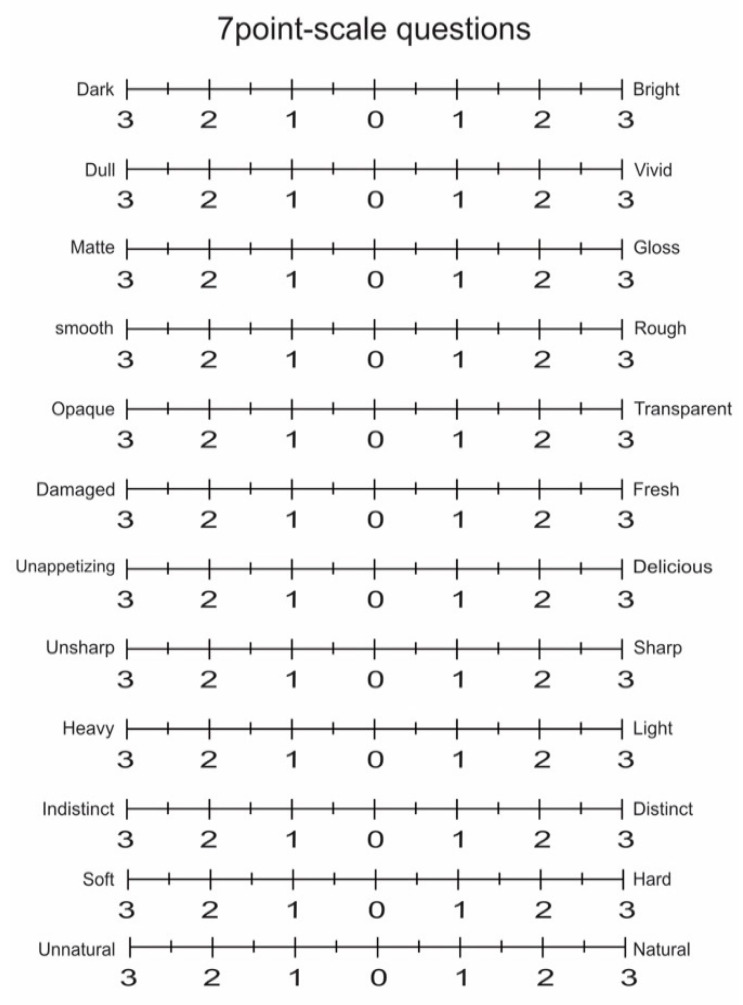
Twelve questions of seven-point scale.

Participants observed a stimulus under all nine viewing conditions using a combination of three spotlight sizes and three illuminance levels. The best viewing condition under which the material appearance of the stimulus was closest to that of the reference condition under natural illumination was then selected. The evaluation of the impression of the stimuli was also conducted for each viewing condition including the reference condition with natural illumination. Participants responded to twelve questions using the seven-point scale shown in Figure 6. The questions included the following items; bright-dark (brightness), vivid-dull (colorfulness), gloss-matte (glossiness), smooth-rough (roughness), transparent-opaque (transparency), fresh-bad (freshness), delicious-awful (delicious looking), sharp-blunt (sharpness), light-heavy (weight), distinct-indistinct (distinctness), hard-soft (hardness) and natural-unnatural (naturalness) [8,18,24]. Direct reports were obtained from the participants after completion of all the trials.

### 2.3. Procedure

Firstly, a participant held and observed a stimulus under natural illumination condition for 10 min in the reference phase. The participant observed the stimuli from various angles by rotating them by hand and memorized the material appearance. After the observation, the participant evaluated the impressions of the stimulus using the seven-point scale. A rest time was set to create a firm object impression and a test phase was started 30 min after the reference phase. The participant then sat in front of a viewing booth and the viewing distance to a stimulus was set an approximately 60 cm. The stimulus was observed and its impressions were evaluated using the seven-point scale questions for one of nine viewing conditions. Then the participant repeated the evaluation for all nine viewing conditions in random order. Upon completion of this task, participants selected a condition for which the appearance of a stimulus was closest to that of the reference condition. Each session consisted of the evaluation of impression under the nine conditions and the fidelity judgment. Three sessions were executed for each of the four stimuli, and twelve sessions (3 repeats × 4 stimuli) were executed in total. Participant produced direct reports after the completion of all sessions.

### 2.4. Participants

Two males and four females participated. The range of their age was 22 to 25 years old (the average was 23.0 years old). All participants had normal visual acuity (binocular vision was 1.0 or better) either naturally or with correction and normal color vision, which was confirmed using the Ishihara color vision test plate and an anomaloscope (OT-II, Neitz). A thorough explanation regarding the purpose and the experimental method was provided to participants and their consent was obtained prior to the experiment.

### 2.5. Analysis

A two-way ANOVA statistical test was used to compare the average frequency of fidelity selection as well as the results of the seven-point scale questions for the reference and test conditions. Multiple comparisons of the Tukey–Kramer test were used to compare each factor including spotlight size, illuminance, stimulus, and participant.

## 3. Results

**Figure 7:**
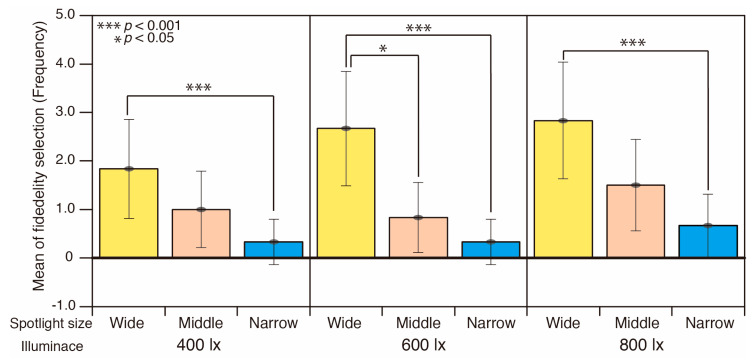
Result of the fidelity selection for each diffuseness and illuminance conditions.

The result of the fidelity selection is shown in Figure 7. The selection of a stimulus closest to the appearance of the reference was examined. The horizontal axis represents the nine viewing conditions and the vertical axis represents the frequency that each condition was chosen. Each bar corresponds to the average result of all participants and food samples. The error bars represent the standard deviation. The wide spotlight condition at 800 lx was chosen most frequently followed by 600 lx and 400 lx, the middle size spotlight condition at 800 lx, 600 lx and 400 lx, then the narrow spotlight condition at 800 lx, 600 lx and 400 lx. There are significant differences between wide and narrow (*p* < 0.05) spotlight conditions at 800 lx, wide and narrow (*p* < 0.001), wide and middle (*p* < 0.05) at 600 lx, and wide and narrow (*p* < 0.001) at 400 lx. There are no significant differences between the illuminance levels (F = 0.439). These results suggest that the illuminance level did not influence the material appearance, but spotlight size with different diffuseness has a large impact.

**Figure 8:**
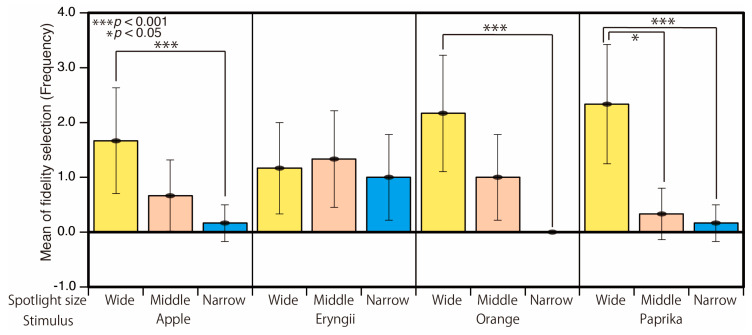
Result of fidelity selection for each stimulus.

In Figure 8, we compared the results for each food sample stimulus. The horizontal axis represents the spotlight size conditions for each stimulus and the vertical axis represents the frequency of fidelity selection. Each bar corresponds to the average of all participants and all illuminance levels. The error bars represent the standard deviation. The wide spotlight conditions are chosen more frequently than the narrow spotlight conditions for Apple (*p* < 0.05), Orange (*p* < 0.001), and Paprika (*p* < 0.001). There is also a significant difference between the middle and narrow condition (*p* < 0.001) for Paprika. However, no significant differences were found for Eryngii.

**Figure 9:**
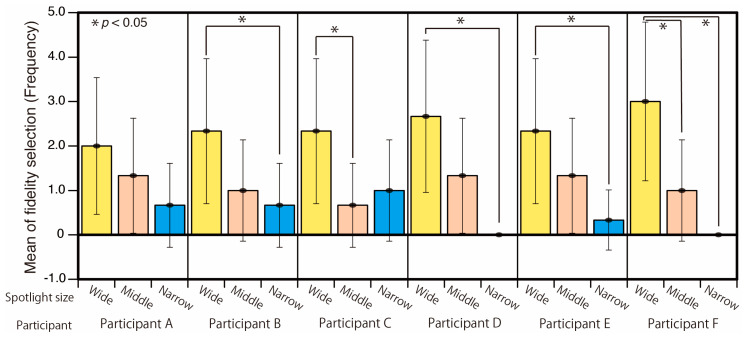
Result of fidelity selection for each participant.

In Figure 9, we compare the results for individual participants. The horizontal axis represents the spotlight size conditions for each participant, and the vertical axis represents the frequency of fidelity selection. Each bar corresponds to the average of all stimuli and all illuminance levels. The wide spotlight condition was chosen most often by the participants. There are significant differences between the wide and narrow conditions for participant B, D, E, and F (*p* < 0.05), and between the wide and middle conditions for participant C and F (*p* < 0.05).

**Figure 10:**
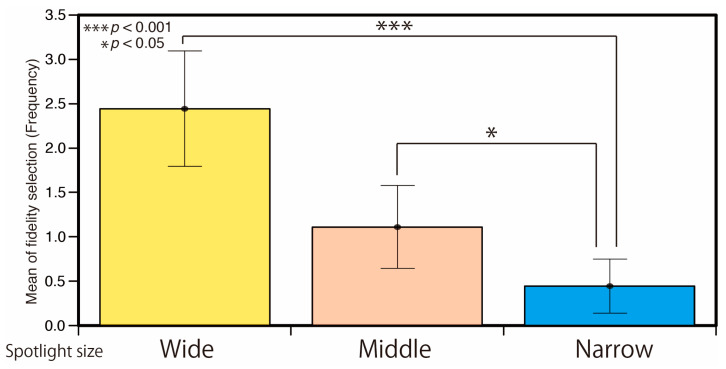
Result of fidelity selection for each spotlight size condition.

Figure 10 shows the result of fidelity selection for each spotlight size. The error bars show the standard deviation. They are the average results of all illuminance levels, stimuli, and participants. The appearance of material was closest to the reference condition for the wide spotlight size condition. There are significant differences between the wide and narrow conditions (*p* < 0.001) and between the middle and narrow conditions (*p* < 0.05).

**Figure 11:**
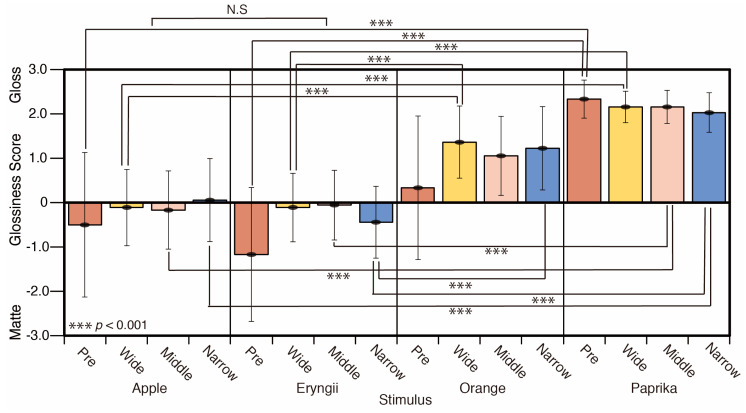
Glossiness scores.

The glossiness scores are presented in Figure 11. The vertical axis represents the seven-point scale score. Each bar corresponds to the average result of all participants and the illuminance levels. The error bars represent the standard deviations. In the reference condition (Pre), the impression of Apple and Eryngii is more matte and that of Orange and Paprika is glossier. The impression of Eryngii and Orange became glossier under spotlight conditions. However, the score of the reference condition (Pre) for Apple is not constant and has large standard deviations. The impression of Paprika is very glossy in all conditions. There are significant differences in glossiness between all combinations except for Apple and Eryngii (*p* < 0.001) when considering the average of all conditions. In the comparison of each spotlight size, significant differences are also detected between apple and orange (*p* < 0.001), Apple and Paprika (*p* < 0.001), Eryngii and Paprika (*p* < 0.001), Eryngii and Paprika in wide (*p* < 0.001), Apple and Paprika (p < 0.001), Eryngii and Paprika (*p* < 0.001) in middle, and Apple and Paprika (*p* < 0.001), Eryngii and Orange (*p* < 0.001), and Eryngii and Paprika (*p* < 0.001) in narrow (*p* < 0.001) spotlight conditions (F = 33.67).

**Figure 12:**
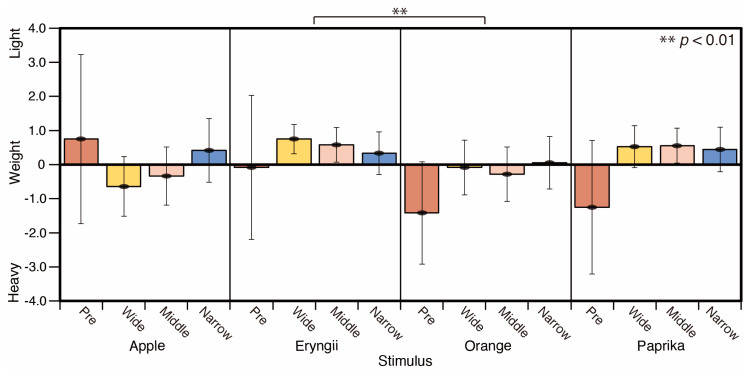
Weight scores.

As shown in Figure 12, Apple appears to be heavier and Eryngii, Orange, and Paprika appears lighter in all spotlight conditions compared to the reference condition (Pre). There are significant differences between the weight score for Eryngii and Orange (*p* < 0.01) in the average of all lighting conditions (F = 3.189). It seems that the narrow spotlight eliminated the heavyweight appearance of Apple.

**Figure 13:**
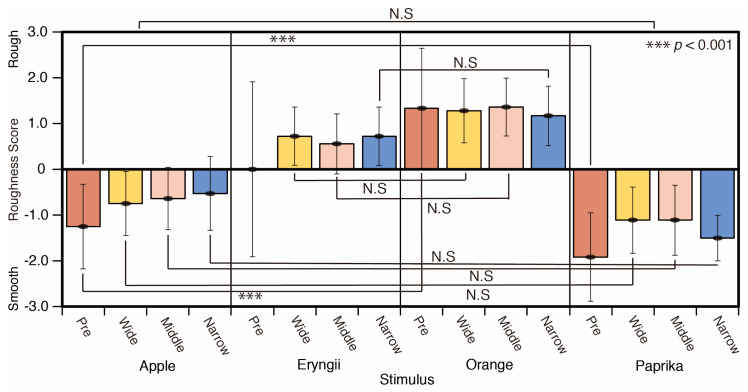
Roughness scores and stimuli.

The roughness scores are summarized in Figure 13. The surface of Apple and Paprika appeared smooth, whereas that of Eryngii and Orange appeared rough. There are significant differences between all combinations except for Apple and Paprika for the average of all lighting conditions (*p* < 0.001). In the comparison of each spotlight size, significant differences are detected between Apple and Orange in the reference phase (pre) (*p* < 0.001), Apple and Eryngii (*p* < 0.001), Apple and Orange (*p* < 0.001), Eryngii and Paprika (*p* < 0.001), and Orange and Paprika (*p* < 0.001) for the wide and middle spotlight condition, for all combinations except for Apple and Paprika in narrow spotlight conditions (*p* < 0.001) (F = 45.55).

**Figure 14:**
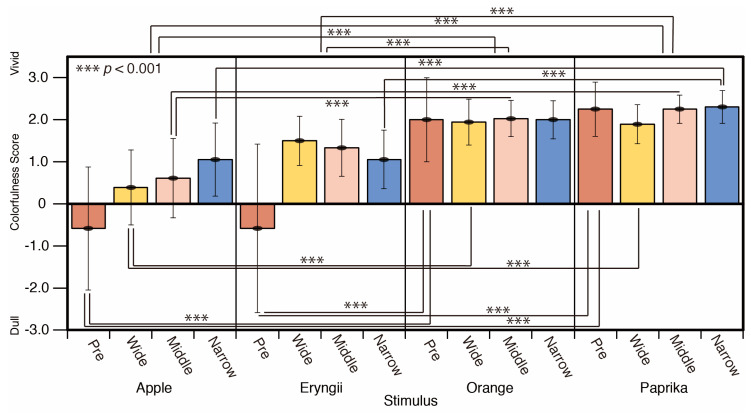
Colorfulness scores and Stimuli.

A comparison of the colorfulness scores by stimulus type is shown in Figure 14. The colorfulness of Orange and Paprika do not show for the different lighting conditions. Apple and Eryngii appeared more vivid under spotlight conditions compared to the reference phase (Pre). There are significant differences in colorfulness between Apple and Eryngii (*p* < 0.001), Apple and Orange (*p* < 0.001), Eryngii and Orange (*p* < 0.001), and Eryngii and Paprika (*p* < 0.001). In the comparison of each spotlight size, significant differences are observed between Apple and Orange (*p* < 0.001), Apple and Paprika (*p* < 0.001), Eryngii and Orange (*p* < 0.001), and Eryngii and Paprika (*p* < 0.001) for the reference phase (Pre) and narrow spotlight condition, Apple and Orange (*p* < 0.001), Apple and Paprika (*p* < 0.001) in wide and middle spotlight condition (F = 20.67).

**Figure 15:**
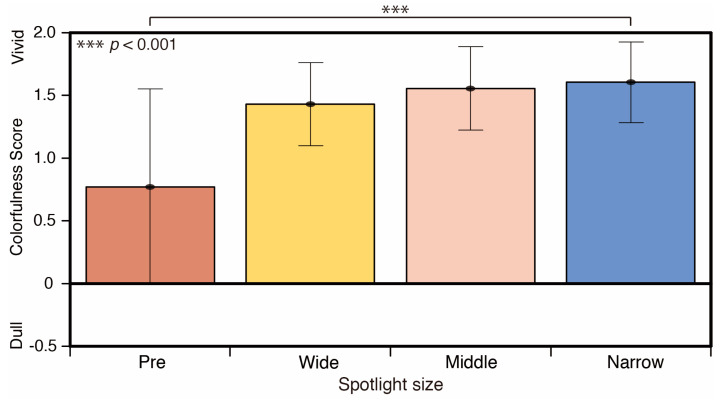
Colorfulness scores and Spotlight size.

A comparison of the colorfulness scores by spotlight size is shown in Figure 15. The score for the spotlight conditions is more vivid than that of the reference phase (Pre). There was a significant difference between the reference phase (Pre) and the narrow spotlight condition (*p* < 0.001) (F = 2.477).

**Figure 16:**
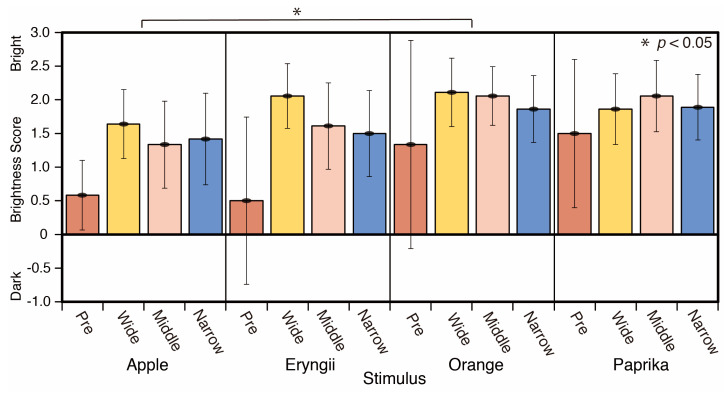
Brightness scores and stimulus.

A comparison of the brightness scores by stimulus is shown in Figure 16. The score of Orange was brighter than Apple. There are significant differences between Apple and Orange (*p* < 0.05) in the average of all spotlight conditions (F = 3.407).

**Figure 17:**
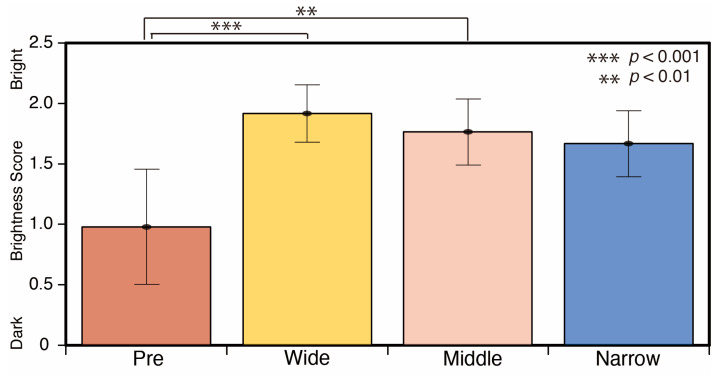
Brightness and spotlight size scores.

A comparison of the brightness scores based on spotlight size is shown in Figure 17. The score of spotlight conditions was brighter than that of the reference phase (Pre). There were significant differences between the reference phase (Pre) and wide (*p* < 0.001), and the reference phase (Pre) and middle spotlight conditions (*p* < 0.01) (F = 4.372).

**Figure 18:**
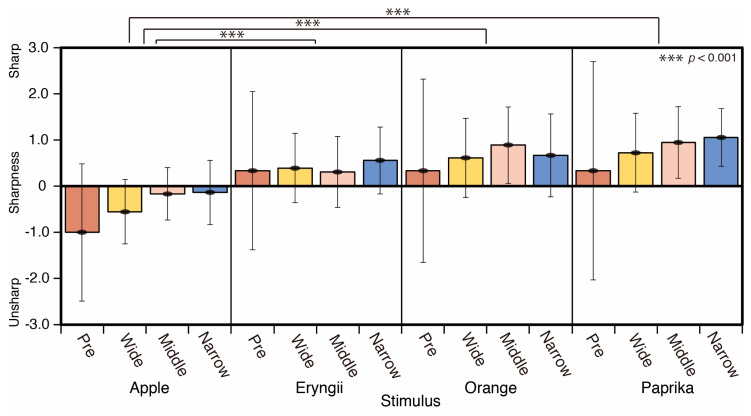
Sharpness scores.

The sharpness scores are shown in Figure 18. Orange, Eryngii, and Paprika appeared sharper than Apple. Sharpness was correlated to a shift of spotlight changes. There are significant differences between Apple andEryngii (*p* < 0.001), Apple and Orange (*p* < 0.001), and Apple and Paprika for the average of all spotlight conditions (*p* < 0.001) (F = 7.095).

**Figure 19:**
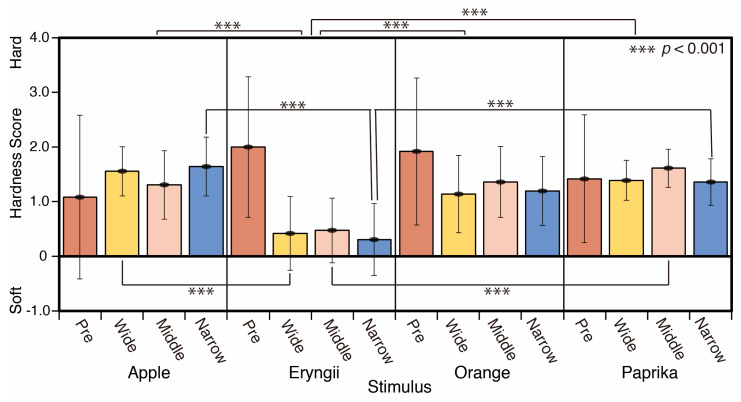
Hardness scores.

The results for the hardness scores are shown in Figure 19. Eryngii appeared softer than Apple, Orange, and Paprika under the three spotlight conditions. There are significant differences between Apple and Eryngii (*p* < 0.001), Eryngii and Orange (*p* < 0.001), and Eryngii and Paprika (*p* < 0.001) for the average of all spotlight conditions. There is a significant difference between Apple and Eryngii (*p* < 0.001) in wide, Eryngii and Paprika (*p* < 0.001) in middle, Apple and Eryngii (*p* < 0.001), and Eryngii and Paprika (*p* < 0.001) for narrow spotlight condition (F = 8.27).

According to the results of the direct reports, participants did not recognize the difference in illuminance conditions, but they were able to distinguish between the change in spotlight size for each observation. All the participants determined that the narrow spotlight condition produced strong shade and shadow in the observed scene.

## 4. Discussion

**Figure 20:**
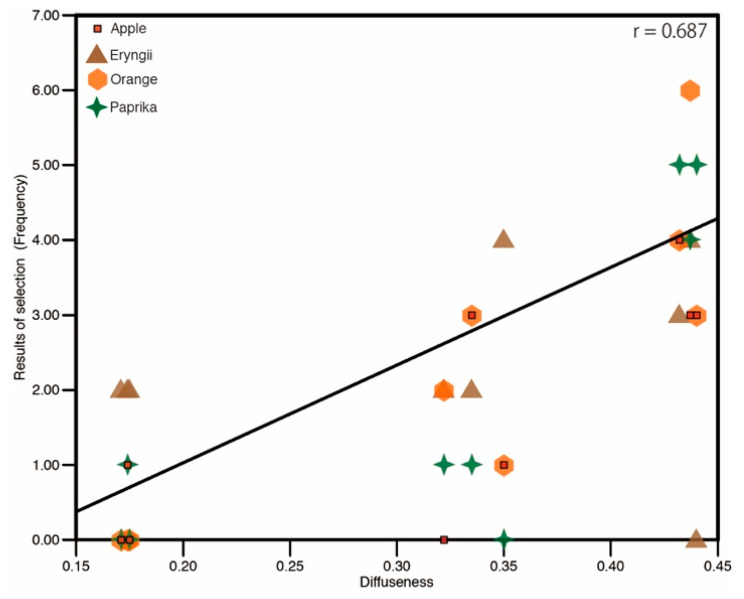
Correlation between diffuseness and fidelity selection.

**Figure 21:**
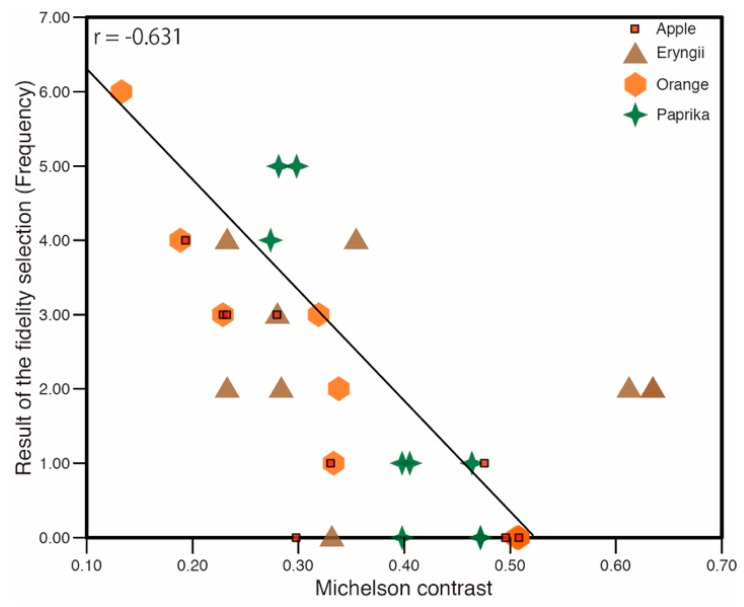
Correlation between luminance contrast and fidelity selection.

**Figure 22:**
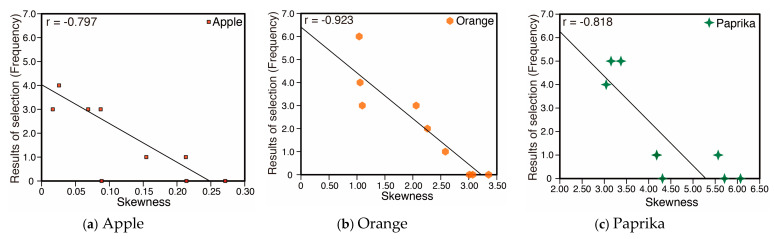
Correlation between the skewness of luminance histogram and fidelity selection. (**a**) Apple, (**b**) Orange, (**c**) Paprika.

Our results show that observers were able to make a judgment on fidelity selection even if the range of diffuseness change due to the fact that the spotlight that was tested departs from the diffuseness level of natural illumination because of technical restrictions. In the present study, the result for fidelity selection shows that a wide spotlight can comprehensively reproduce material appearance that is closest to that of natural illumination among the tested conditions. The main difference in spotlight size was diffuseness and wide spotlight had high diffuseness. This result is in agreement with that of previous studies that identified a relation between object surface perception and the diffuseness of the illumination source [10,17]. Figure 20 shows the relation between the frequency of fidelity selection and the diffuseness of lighting. Although there are deviations due to differences in stimulus, Pearson’s rank correlation shows a moderately positive correlation (r = 0.687). This suggests that samples under lighting with high diffuseness show higher fidelity compared to material appearance under natural illumination. As shown in Figure 3 and Figure 4, the image statistics such as the contrast and skewness of the luminance histogram change according to the change in the spotlight size or diffuseness. Figure 21 shows the relation between the frequency of fidelity selection and the luminance contrast of stimuli. Pearson’s rank correlation shows a moderately negative correlation (r = −0.631) indicating that a lower contrast resulted in higher fidelity. Figure 22 shows the relation between the frequency of fidelity selection and the skewness of luminance histogram for Apple, Orange, and Paprika. Pearson’s rank correlation shows high negative correlations for Apple, Orange, and Paprika, but the result is not significant for Eryngii (r = −0.136). These results suggest that the change in luminance distribution on the object surface due to diffuseness of illumination could be a major factor that influences material appearance under different spotlights. The skewness would not be applicable for all conditions but could be a strong factor for particular objects and materials.

However, illuminance did not influence judgment in the present study. This could be because light adaptation occurred easily and participants were not aware of illuminance changes. The results for fidelity selection and the seven-point scale questions suggest that the characteristics of the material’s surface largely influences its appearance. In particular, the influence of macro-texture and micro-texture are different.

**Figure 23:**
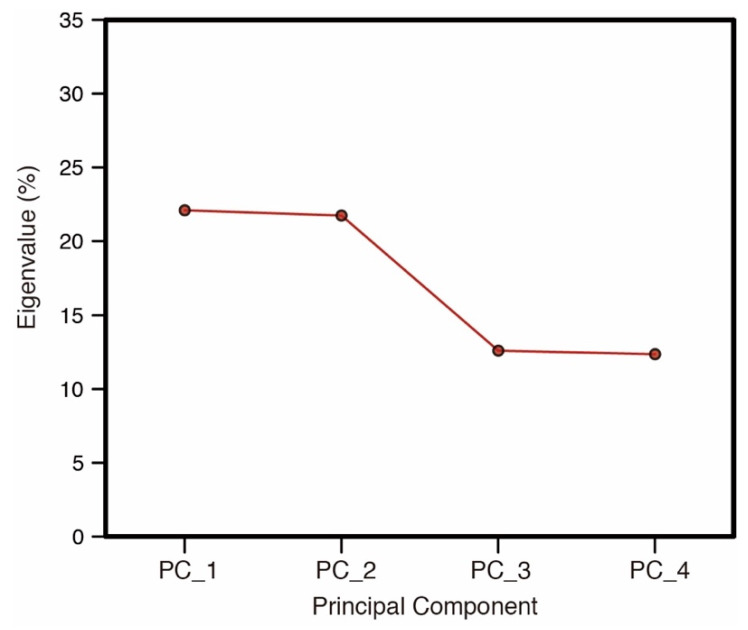
Eigenvalue of each principal component.

**Figure 24:**
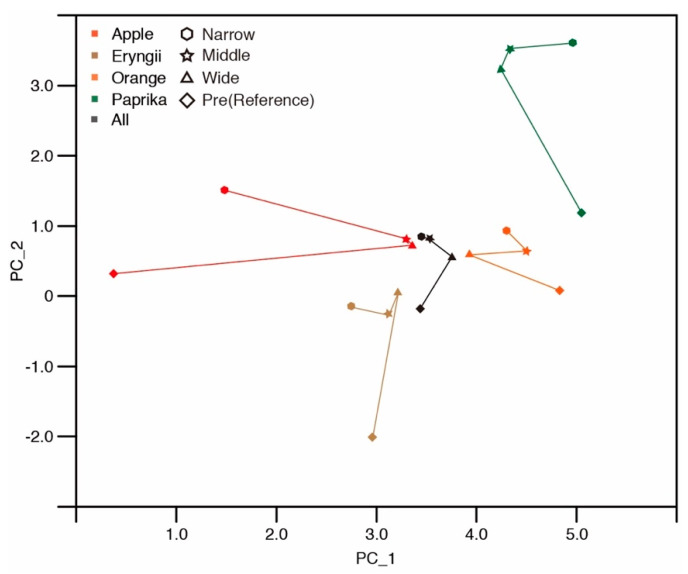
Distribution of the median of spotlight conditions on principal components.

To further examine the characteristics of differences in impression, we applied principal component analysis [8,9] to the results of seven-point scale questions. Table 2 shows the results of this analysis. The factor loadings of the first two principal components indicate strong positive loadings of naturalness, freshness, delicious looking, colorfulness, brightness, and clearness on the first principal component (PC_1), and the positive loadings of clearness, delicious looking, colorfulness, brightness. In addition, negative loadings of transparency, and weight were indicated on the second principal component (PC_2). Figure 23 shows the eigenvalues for the analysis. The first four principal components represent 68.5% of the total information. In particular, PC_1 and PC_2 occupied 43.7%, and they have the largest number of eigenvalues. The distribution of the median of each lighting condition is shown in Figure 24. The x- and y-axis represents PC_1 and PC_2, respectively. The median of all stimuli shows an increase in the PC_2 values in accordance with the decrease of the diffuseness of illumination. This suggests that PC_2 corresponds to the diffuseness factor. Only the wide spotlight condition for Eryngii did not follow this trend. The results for PC_2 are consistent with the result for the three spotlight conditions on fidelity selection for each stimulus (Figure 8).

PC_2 was correlated with spotlight size changes for different diffuseness. This reveals that not only fidelity selection but also the impression of the stimuli was influenced by the spotlight size or the diffuseness of the illumination. These results again suggest that diffuseness could be one of the strong factors that influence material appearance. For example, significant differences were observed in brightness and colorfulness for different spotlight sizes. The eigenvalues of brightness and colorfulness for PC_1 and PC_2 are relatively high, suggesting that these two factors were influenced by the diffusivity of illumination.

The diffuseness of illumination is not the only factor that influences material appearance. The original properties of objects such as their shape and materials are crucial to their appearance. The result of the seven-point scale questions suggests that the surface texture of objects influence the appearance of glossiness. Stimuli with small macro-texture like Apple and Paprika did not exhibit changes in their glossiness among the different spotlight conditions. However, stimuli with a large macro-texture like Eryngii and Orange exhibited an increase in their glossiness. The result of the seven-point scale survey suggests that micro-texture also influences the appearance of colorfulness, roughness, brightness, and weight. Gloss stimuli like Orange and Paprika did not show changes in colorfulness due to spotlights. However, matte stimuli like Apple and Eryngii showed colorfulness changes. Apple and Eryngii appeared more vivid compared to the reference observation. The colorfulness of Apple increased when the spotlight size was narrowed, whereas that of Eryngii decreased when the spotlight size was narrowed. The score for Eryngii was best for the wide spotlight, suggesting that there exists a diffuseness level that shows the best colorfulness between the wide spotlight and natural illumination. Gloss stimuli like Orange and Paprika did not show changes in roughness between the reference and spotlights, whereas matte stimuli such as Apple and Eryngii exhibited roughness changes. The brightness of all stimuli was increased by the spotlight compared to the reference phase. However, the magnitude of the increase was much larger for matte stimuli like Apple and Eryngii compared to those of gloss and smooth stimuli such as Orange and Paprika. Gloss stimuli like Orange and Paprika showed a large difference in weight between the reference and spotlights, but matte stimuli such as Apple and Eryngii did not. The results for sharpness and hardness suggest that appearance judgments depend on the unique stimulus character. Apple appeared unsharp, and Eryngii appeared soft, which are different trends compared to the other stimuli. These results suggest that the influence of the micro-texture of objects on material appearance judgment could be significant. However, the relationship and the reason for the difference are not clear at the present stage. Nonetheless, the results of the principal component analysis suggest that Principal Component 1 (PC_1) and Principal Component 2 (PC_2) could be classified as the material appearance and illumination. The results obtained support previous research showing that similar information about materials can be classified based on our impression [8]. The influence of the spotlight size and diffuseness are material dependent and further investigation is needed to clarify the relationship between each impression and the micro- and macro-texture.

## 5. Conclusions

In the present study, we showed that the fidelity of appearance that is closest to that of natural illumination is obtained under a wide spotlight. We did not determine the influence of the illuminance levels on judgment. This suggests that the diffuseness of illumination influences the material appearance of objects. The results for the seven-point scales survey revealed differences between the samples but little difference between the lighting conditions including the different illuminance levels and spotlight sizes. The result of principal component analysis suggests that the spotlight size influences the impression of objects. These results indicate that lighting with relativity high diffuseness would be adequate for the reproduction of material appearance under spotlight illumination with some adjustments considering the characteristics of the object and material surface condition such as macro and micro-textures. Although more investigation is required, we showed that it is possible to set an appropriate lighting condition to realize material appearance similar to the impression learned using visual and tactile information under natural illumination.

## Figures and Tables

**Table 1:** Illuminance, spotlight size, and diffuseness for different viewing conditions.

Illuminance (lx)	Ref.(Ave.)	800	600	400	800	600	400	800	600	400
**Spotlight size**	Natural	Wide	Wide	Wide	Middle	Middle	Middle	Narrow	Narrow	Narrow
**Diffuseness (*D*_Cuttle_)**	0.554	0.437	0.432	0.440	0.335	0.322	0.350	0.174	0.171	0.175

**Table 2:** Factor loadings of the first four principal components. (after varimax rotation).

Item	PC_1	PC_2	PC_3	PC_4
Naturalness	0.761	−0.142	−0.136	−0.175
Freshness	0.754	0.005	0.049	0.389
Delicious looking	0.713	0.477	0.004	−0.004
Colorfulness	0.631	0.465	0.191	0.271
Brightness	0.576	0.446	0.206	0.33
Transparency	−0.1	−0.903	−0.089	0.13
Clearness	0.432	0.742	0.098	0.247
Weight	0.051	−0.728	0.02	−0.077
Glossiness	0.095	−0.042	0.837	0.319
Sharpness	−0.072	0.157	0.826	−0.277
Hardness	0	−0.016	0.003	0.881
Roughness	0.212	0.122	0.006	0.272
